# Findings from the First Year of a Federally Funded, Direct-to-Consumer HIV Self-Test Distribution Program — United States, March 2023–March 2024

**DOI:** 10.15585/mmwr.mm7324a4

**Published:** 2024-06-20

**Authors:** Travis Sanchez, Robin J. MacGowan, Jennifer Hecht, Jessica M. Keralis, Lucinda Ackah-Toffey, Avery Bourbeau, Ruth Dana, Emily A. Lilo, Revae S. Downey, Hannah Getachew-Smith, Marissa Hannah, Rachel Valencia, Eli Krebs, Emily S. Pingel, Jennie Johnston Gayden, Jenna Norelli, Zavier Mason, Jennifer Mahn, Natalie Cramer, Robert Bole, Patrick Sullivan, Anuli N. Nwaohiri, Jo Ellen Stryker, Athena P. Kourtis, Elizabeth A. DiNenno, Robyn Neblett Fanfair, Jonathan H. Mermin, Kevin P. Delaney

**Affiliations:** ^1^Emory University, Atlanta, Georgia; ^2^Division of HIV Prevention, National Center for HIV, Viral Hepatitis, STD, and TB Prevention, CDC; ^3^Building Healthy Online Communities, San Francisco, California; ^4^DLH, Washington, DC; ^5^NASTAD, Washington, DC; ^6^Signal Group, Inc. Washington, DC; ^7^National Center for HIV, Viral Hepatitis, STD, and TB Prevention, CDC.

**Summary**

**What is already known about this topic?**

HIV self-testing is a cost-effective method for expanding testing access to persons with barriers to other in-person HIV testing options.

**What is added by this report?**

During March 14, 2023–March 13, 2024, a CDC-funded program delivered approximately 440,000 mailed HIV self-tests (HIVSTs) to U.S. residents, including those disproportionately affected by HIV, 24.1% of whom had never previously received testing; 1.9% reported receiving a positive HIV test result. Many sought additional clinical services shortly after receiving their HIVSTs.

**What are the implications for public health practice?**

Clinicians, community organizations, and public health officials should be aware of HIVST programs, initiate discussions about HIV testing conducted outside their clinics or offices, and initiate follow-up services for persons who report a positive or negative HIVST result.

## Abstract

In September 2022, CDC funded a nationwide program, Together TakeMeHome (TTMH), to expand distribution of HIV self-tests (HIVSTs) directly to consumers by mail through an online ordering portal. To publicize the availability of HIVSTs to priority audiences, particularly those disproportionately affected by HIV, CDC promoted this program through established partnerships and tailored resources from its Let’s Stop HIV Together social marketing campaign. The online portal launched March 14, 2023, and through March 13, 2024, distributed 443,813 tests to 219,360 persons. Among 169,623 persons who answered at least one question on a postorder questionnaire, 67.9% of respondents were from priority audiences, 24.1% had never previously received testing for HIV, and 24.8% had not received testing in the past year. Among the subset of participants who initiated a follow-up survey, 88.3% used an HIVST themselves, 27.1% gave away an HIVST, 11.7% accessed additional preventive services, and 1.9% reported a new positive HIVST result. Mailed HIVST distribution can quickly reach large numbers of persons who have never received testing for HIV or have not received testing as often as is recommended. TTMH can help to achieve the goal of diagnosing HIV as early as possible and provides a path to other HIV prevention and care services. Clinicians, community organizations, and public health officials should be aware of HIVST programs, initiate discussions about HIV testing conducted outside their clinics or offices, and initiate follow-up services for persons who report a positive or negative HIVST result.

## Introduction

Distributing HIV self-tests (HIVSTs) is an effective (*1*) and cost-effective (*2*,*3*) means of providing HIV testing for persons who could be living with undiagnosed HIV infection, including gay, bisexual, and other men who have sex with men (MSM). HIV self-testing is recommended as a testing strategy for diverse populations worldwide (*4*) and is considered an important strategy in the Ending the HIV Epidemic in the US (EHE) initiative (*5*,*6*). HIVSTs can also facilitate access to antiretroviral treatment, HIV preexposure prophylaxis (PrEP), and other prevention services (*1*,*7*). In September 2022, CDC funded Emory University and its partners, Building Healthy Online Communities (BHOC), OraSure Technologies, Signal Group, and NASTAD to expand a nationwide HIVST distribution program with a goal of distributing at least 1 million HIVSTs over 5 years.* CDC’s national Let’s Stop HIV Together campaign^†^ designed tailored promotions for priority audiences (i.e., persons disproportionately affected by HIV, including MSM, transgender women [of any race], and cisgender non-Hispanic Black or African American [Black] women)^§^ to order HIVSTs. This report describes the first year of the program, Together TakeMeHome (TTMH),^¶^ which launched March 14, 2023.

## Methods

The TTMH program mails up to two Food and Drug Administration–approved HIVSTs at no cost to the participant to persons aged ≥17 years in the United States (including Puerto Rico) who place an order through the site, irrespective of health insurance or immigration status. After ordering and indicating whether they are willing to receive future communication, program participants are asked to describe themselves by answering a brief postorder questionnaire. Participants who opted into future communications received follow-up evaluation surveys 10 and 60 days after their order. Participants may reorder HIVSTs every 90 days. Demographic characteristics and previous HIV testing history were summarized for all persons who provided any postorder information after their first order from the program.** Among those who responded to a follow-up evaluation survey, percentages^††^ of respondents who used an HIVST themselves, shared an HIVST with others, accessed other HIV and sexually transmitted infection (STI) prevention services, or received a positive HIV test result on an HIVST they used, were stratified by participant demographic characteristics and geographic information collected to process the order. All analyses were conducted using SAS software (version 9.4; SAS Institute). These programmatic activities were reviewed by CDC, deemed not research, and conducted consistent with applicable federal law and CDC policy.^§§^

## Results

During March 14, 2023–March 13, 2024, TTMH distributed 443,183 tests to 219,360 persons, including 16,365 (7.5%) who ordered from the program more than once. Overall, 86.0% of orders were for two HIVSTs; 14.0% were for only one test. Among 169,623 (77.3%) persons who completed at least one postorder question, the majority were aged <35 years (60.1%), cisgender men (71.5%), identified as Hispanic or Latino (Hispanic) or Black (54.7%), and lived in large central or fringe metropolitan counties (59.5%); one half lived in EHE focus jurisdictions^¶¶^ (Table 1). Approximately two thirds (65.3%) of orders were placed by persons recruited from messaging tailored for gay and bisexual men on lesbian, gay, bisexual, transgender, and queer-plus (LGBTQ+)–focused dating apps. Overall, 54.6% of orders could be attributed to one of the priority audiences, and an additional 13.3% with missing information came to the program from promotions on an LBGTQ+ dating app. Few persons (7.3%) were currently using PrEP or had received an HIV diagnosis (2.4%); a message generated on the TTMH website encouraged these persons to give their HIVSTs to others who might benefit more from the test. Overall, 24.1% of persons had never previously received testing for HIV and 24.8% had not received testing within the previous year. The proportion who had never received testing varied substantially by age (from 45.4% among those aged 17–24 years to 13.2% among those aged 35–44 years), gender identity (from 35.2% among transgender men to 23.5% among cisgender men), and county population density (from approximately 31.0% in noncore and micropolitan [more rural] areas to 20.7% in large central metropolitan areas [urban centers or cities]). Among priority audiences, the highest percentage of persons who reported receiving testing during the previous 12 months (58.4%) were Black cisgender MSM.

**TABLE 1:** HIV testing history and demographic characteristics reported by persons who ordered at least one HIV self-test and answered at least one postorder survey question* (N = 169,623) — Together TakeMeHome HIV self-test distribution program, United States, March 14, 2023–March 13, 2024

Characteristic	Total, no. (column %)	History of HIV testing, no. (row %)			
		Within past year	≥1 year ago	Never	Missing
**Total**	**169,623 (100.0)**	**76,790 (45.3)**	**42,122 (24.8)**	**40,846 (24.1)**	**9,865 (5.8)**
**Age group, yrs**					
17–24	**43,148 (25.4)**	16,654 (38.6)	4,827 (11.2)	19,569 (45.4)	2,098 (4.9)
25–34	**58,903 (34.7)**	29,605 (50.3)	14,138 (24.0)	11,820 (20.1)	3,340 (5.7)
35–44	**36,700 (21.6)**	17,854 (48.6)	11,787 (32.1)	4,847 (13.2)	2,212 (6.0)
45–54	**17,803 (10.5)**	7,494 (42.1)	6,721 (37.8)	2,440 (13.7)	1,148 (6.4)
≥55	**13,061 (7.7)**	5,178 (39.6)	4,648 (35.6)	2,169 (16.6)	1,066 (8.2)
Missing	**8 (0)**	5 (62.5)	1 (12.5)	1 (12.5)	1 (12.5)
**Gender^†^**					
Cisgender man	**121,334 (71.5)**	58,731 (48.4)	27,360 (22.5)	28,501 (23.5)	6,742 (5.6)
Cisgender woman	**30,374 (17.9)**	10,308 (33.9)	11,217 (36.9)	7,681 (25.3)	1,168 (3.8)
Transgender man or transmasculine	**1,972 (1.2)**	825 (41.8)	372 (18.9)	694 (35.2)	81 (4.1)
Transgender woman or transfeminine	**4,027 (2.4)**	2,003 (49.7)	736 (18.3)	1,041 (25.9)	247 (6.1)
Another gender identity	**9,503 (5.6)**	4,271 (44.9)	2,081 (21.9)	2,577 (27.1)	574 (6.0)
Missing	**2,413 (1.4)**	652 (27.0)	356 (14.8)	352 (14.6)	1,053 (43.6)
**Race and ethnicity^§^**					
AI/AN	**1,833 (1.1)**	828 (45.2)	415 (22.6)	484 (26.4)	106 (5.8)
Asian	**7,591 (4.5)**	3,697 (48.7)	1,404 (18.5)	2,104 (27.7)	386 (5.1)
Black or African American	**41,271 (24.3)**	20,175 (48.9)	10,000 (24.2)	8,868 (21.5)	2,228 (5.4)
NH/PI	**807 (0.5)**	362 (44.9)	179 (22.2)	227 (28.1)	39 (4.8)
White	**58,673 (34.6)**	25,209 (43.0)	16,020 (27.3)	14,668 (25.0)	2,776 (4.7)
Hispanic or Latino	**51,588 (30.4)**	23,104 (44.8)	12,246 (23.7)	12,913 (25.0)	3,325 (6.4)
Multiracial and other	**6,254 (3.7)**	2,908 (46.5)	1,552 (24.8)	1,284 (20.5)	510 (8.2)
Missing	**1,606 (1.0)**	507 (31.6)	306 (19.1)	298 (18.6)	495 (30.8)
**Population density^¶^**					
Large central metropolitan	**66,871 (39.4)**	33,611 (50.3)	15,153 (22.7)	13,839 (20.7)	4,268 (6.4)
Large fringe metropolitan	**34,025 (20.1)**	15,426 (45.3)	8,409 (24.7)	8,220 (24.2)	1,970 (5.8)
Medium metropolitan	**33,587 (19.8)**	14,728 (43.9)	8,306 (24.7)	8,712 (25.9)	1,841 (5.5)
Small metropolitan	**13,327 (7.9)**	5,491 (41.2)	3,351 (25.1)	3,819 (28.7)	666 (5.0)
Micropolitan	**10,744 (6.3)**	4,075 (37.9)	2,807 (26.1)	3,323 (30.9)	539 (5.0)
Noncore	**5,687 (3.4)**	2,034 (35.8)	1,579 (27.8)	1,780 (31.3)	294 (5.2)
Missing	**5,382 (3.2)**	1,425 (26.5)	2,517 (46.8)	1,153 (21.4)	287 (5.3)
EHE jurisdiction**					
Yes	**84,661 (49.9)**	40,553 (47.9)	21,027 (24.8)	18,790 (22.2)	5,191 (6.1)
No	**84,962 (50.1)**	36,237 (42.7)	21,995 (25.9)	22,056 (26.0)	4,674 (5.5)
**Recruitment source^††^**					
LGBTQ+ dating app	**110,799 (65.3)**	54,629 (49.3)	24,152 (21.8)	25,279 (22.8)	6,739 (6.1)
Other dating app or website	**5,710 (3.4)**	2,505 (43.9)	1,744 (30.5)	1,231 (21.6)	230 (4.0)
Social media	**9,993 (5.9)**	3,238 (32.4)	4,168 (41.7)	2,056 (20.6)	531 (5.3)
Other partner promotions	**16,698 (9.8)**	6,893 (41.3)	4,243 (25.4)	4,605 (27.6)	957 (5.7)
Search engine marketing or web search	**13,051 (7.7)**	4,542 (34.8)	3,855 (29.5)	3,997 (30.6)	657 (5.0)
Direct link	**7,869 (0.1)**	2,724 (34.6)	2,297 (29.2)	2,402 (30.5)	446 (5.7)
Other	**378 (0.2)**	116 (30.7)	163 (43.1)	85 (22.5)	14 (3.7)
Missing	**5,125 (3.0)**	2,143 (41.8)	1,500 (29.3)	1,191 (23.2)	291 (5.7)
**Priority audience^§§^**					
No^¶¶^	**46,043 (27.1)**	14,416 (31.3)	14,160 (30.8)	15,528 (33.7)	1,939 (4.2)
Yes (all with enough information to be categorized)	**92,655 (54.6)**	49,610 (53.5)	21,128 (22.8)	19,121 (20.6)	2,796 (3.0)
Yes, cisgender MSM overall	**75,295 (44.4)**	41,649 (55.3)	15,858 (21.1)	15,691 (20.8)	2,097 (2.8)
Yes, Hispanic cisgender MSM	**24,856 (14.7)**	13,742 (55.3)	4,846 (19.5)	5,481 (22.1)	787 (3.2)
Yes, Black or African American cisgender MSM	**13,272 (7.8)**	7,755 (58.4)	2,194 (16.5)	2,870 (21.6)	453 (3.4)
Yes, transgender women or transfeminine	**4,027 (2.4)**	2,003 (49.7)	736 (18.3)	1,041 (25.9)	247 (6.1)
Yes, Black or African American cisgender women	**13,333 (7.9)**	5,958 (44.7)	4,534 (34.0)	2,389 (17.9)	452 (3.4)
Unable to determine audience because of missing information	**30,925 (18.2)**	12,764 (41.3)	6,834 (22.1)	6,197 (20.0)	5,130 (16.6)
Missing information but recruited from LGBTQ+ dating app***	**22,572 (13.3)**	9,955 (44.1)	4,983 (22.1)	4,370 (19.4)	3,264 (14.5)
**PrEP use^†††^**					
Currently on PrEP	**12,447 (7.3)**	—**^§§§^**	—**^§§§^**	—**^§§§^**	—**^§§§^**
Previously on PrEP	**13,994 (8.3)**	11,158 (79.7)	2,812 (20.1)	—**^§§§^**	—**^§§§^**
No	**95,448 (56.3)**	53,117 (55.7)	38,667 (40.5)	3,527 (3.7)	137 (0.1)
Missing	**47,734 (28.1)**	477 (1.0)	245 (0.5)	37,292 (78.1)	9,720 (20.4)
**HIV positive status^¶¶¶^**					
Yes	**4,047 (2.4)**	—****	—****	—****	—****
No	**115,983 (68.4)**	75,195 (64.8)	40,753 (35.1)	0 (—)	35 (0)
Missing	**49,593 (29.2**	1,463 (3.0)	1,328 (2.7)	40,846 (82.4)	5,956 (12.0)

**Abbreviations:** AI/AN = American Indian or Alaska Native; EHE = Ending the HIV Epidemic in the US; LGBTQ+ = lesbian, gay, bisexual, transgender, or queer-plus; MSM = men who have sex with men; NH/PI = Native Hawaiian or Pacific Islander; PrEP = HIV preexposure prophylaxis.

* Data are limited to the subset of persons who started the postorder survey and completed at least one demographic question (169,623; 77% of all persons who placed at least one order). For persons who ordered from the program more than once, demographic data reported at the time the first order was placed are considered and summarized; only the first order is included in the counts reported.

^†^ Gender identity is a composite of reported sex at birth and current gender identity. Cisgender men and cisgender women reported a current gender identity that aligned with their reported sex at birth. Persons who stated their gender identity was transgender man or transmasculine or who reported female sex at birth and current gender of man were classified as transgender men. Persons who stated their gender identity was transgender woman or transfeminine or who reported male sex at birth and current gender of woman were classified as transgender women. Another gender identity includes persons who identified as nonbinary, gender nonconforming, genderqueer, gender fluid, or another gender.

^§^ Persons of Hispanic or Latino (Hispanic) origin might be of any race but are categorized as Hispanic; all racial groups are non-Hispanic. Other categories reported as a single race or as multiracial when more than one race was reported.

^¶^ Zip codes associated with self-test shipping addresses were assigned to county of residence and then summarized using the National Center for Health Statistics population density categorizations. https://www.cdc.gov/nchs/data_access/urban_rural.htm

** Zip codes associated with self-test shipping addresses were assigned to county of residence and then categorized based on whether or not that county is included in one of the EHE jurisdictions. https://www.cdc.gov/ehe/php/jurisdictions-plans/

^††^ The program ordering portal includes analytics that can categorize the source of recruitment to the website. These broad categories include LGBTQ+ dating app (dating apps for both gay and bisexual persons), other dating app or website (dating apps for other men and women), social media (e.g., Facebook and Instagram), other partner promotions (planned promotional campaigns by CDC partners in the Let’s Stop HIV together social media campaign [https://www.cdc.gov/stophivtogether/partnerships/toolkit/hiv-testing-my-way.html]), search engine marketing or web search, or direct link, when the link to the program was typed directly into an Internet browser.

^§§^ For the first year, program marketing was developed for three priority audiences disproportionately affected by HIV: gay, bisexual, and other MSM; transgender women (of any race); and cisgender Black or African American (Black) women. For MSM, imagery was designed specifically to appeal to Black and Hispanic men, but messages were placed on social media and dating apps inclusive of all MSM.

^¶¶^ Transgender men and other gender-diverse persons, cisgender women who did not identify as Black, and men who only reported female or gender-diverse partners were not considered to have been among the priority audiences for marketing the program during the period covered by the report.

*** Persons who did not provide enough demographic or sexual behavior information but could be categorized as arriving at the ordering website from an LGBTQ+ dating app are also considered to have been reached by marketing efforts for priority audiences.

^†††^ Use of HIV preexposure prophylaxis was ascertained through a question that asked about never or no use, current use, use in the previous year but not currently, and use more than a year ago but not currently. In this report, the latter two categories have been combined as any previous PrEP use.

^§§§^ Dashes indicate data are not reported. Because use of PrEP requires receipt of an HIV test, HIV testing history data are summarized only for the group reporting no previous or current PrEP use.

^¶¶¶^ Self-reported HIV-positive status was determined from responses to a question about having ever received a positive HIV test result.

**** Dashes indicate data are not reported. Because program users who report receipt of a positive HIV test result have a history of receiving an HIV test and would not require ongoing retesting, HIV testing history data are not reported for this group.

Among the 14,217 HIVST recipients who responded to at least one question on either the 10- or 60-day follow-up survey, 88.3% reported that they had used an HIVST themselves; 27.1% gave away an HIVST; 11.7% (1,171 of 10,048) accessed additional preventive services (including 4.8% who accessed PrEP services); and 8.6% accessed additional STI testing after ordering from TTMH (Table 2). Sharing HIVSTs with others was more commonly reported by persons aged >35 years; those living in small metropolitan, micropolitan, or noncore counties; and Black cisgender women. Among priority audiences, the largest percentages to access PrEP and STI testing were cisgender MSM (6.5%) and transgender women (11.3%), respectively. Among 7,893 persons who used the HIVST themselves and who had not reported a previous HIV diagnosis, 151 (1.9%) reported receiving a positive test result; nearly every demographic group included at least one person who reported receiving a positive test result. The highest percentages of persons who reported receiving a positive test result were transgender women (3.6%), Black MSM (3.0%), and Hispanic MSM (2.9%); the lowest percentage was reported by Black cisgender women (0.8%).

**TABLE 2:** Characteristics of persons who received and used an HIV self-test and reported one or more outcomes on a follow-up survey (N = 14,217) — Together TakeMeHome HIV self-test distribution program, United States, March 14, 2023–March 13, 2024

Characteristic	no./No. (row %)*					
	HIV self-test use		Other services accessed after receiving self-test			New positive HIV self-test result
	Used themselves	Shared with others	HIV testing	PrEP service	STI testing	
**Total**	**7,793/8,828 (88.3)**	**2,490/9,178 (27.1)**	**1,918/9,194 (20.9)**	**479/10,048 (4.8)**	**797/9,239 (8.6)**	**151/7,893 (1.9)**
**Age group, yrs^†^**						
17–24	1,138/1,245 (91.4)	240/1,286 (18.7)	258/1,294 (19.9)	81/1,461 (5.5)	97/1,295 (7.5)	24/1,153 (2.1)
25–34	2,303/2,543 (90.6)	690/2,637 (26.2)	608/2,645 (23.0)	127/2,884 (4.4)	237/2,656 (8.9)	53/2,335 (2.3)
35–44	2,176/2,442 (89.1)	795/2,535 (31.4)	560/2,532 (22.1)	141/2,747 (5.1)	265/2,549 (10.4)	46/2,201 (2.1)
45–54	1,180/1,376 (85.8)	450/1,431 (31.5)	260/1,431 (18.2)	79/1,539 (5.1)	119/1,438 (8.3)	15/1,195 (1.3)
≥55	996/1,221 (81.6)	315/1,288 (24.5)	232/1,291 (18.0)	51/1,416 (3.6)	79/1,300 (6.1)	13/1,009 (1.3)
**Gender^§^**						
Cisgender man	5,676/6,388 (88.9)	1,659/6,667 (24.9)	1,459/6,674 (22.9)	409/7,270 (5.6)	564/6,710 (8.4)	118/5,755 (2.1)
Cisgender woman	1,457/1,664 (87.6)	611/1,697 (36.0)	274/1,708 (16.0)	14/1,888 (0.7)	142/1,711 (8.3)	17/1,467 (1.2)
Transgender man or transmasculine	78/98 (79.6)	23/102 (22.6)	20/103 (19.4)	14/111 (12.6)	13/104 (12.5)	—^¶^/79 (—^¶^)
Transgender woman or transfeminine	163/178 (91.6)	56/186 (30.1)	51/184 (27.7)	11/206 (5.3)	21/186 (11.3)	—^¶^/165 (—^¶^)
Another gender identity	379/457 (82.9)	129/481 (26.8)	103/480 (21.5)	30/523 (5.7)	51/483 (10.6)	—^¶^/387 (—^¶^)
Missing	40/43 (93.0)	12/45 (26.7)	11/45 (24.4)	1/50 (2.0)	6/45 (13.3)	—^¶^/40(—^¶^)
Race and ethnicity**						
AI/AN	82/88 (93.2)	36/90 (40.0)	21/90 (23.3)	—^¶^/104 (—^¶^)	12/91 (13.2)	—^¶^/83 (—^¶^)
Asian	316/343 (92.1)	64/346 (18.5)	97/346 (28.0)	23/369 (6.2)	34/346 (9.8)	—^¶^/318 (—^¶^)
Black or African American	1,695/1,916 (88.5)	574/1,998 (28.7)	409/1,997 (20.5)	74/2,175 (3.4)	164/2,007 (8.2)	27/1,717 (1.6)
NH/PI	27/32 (84.4)	10/33 (30.3)	15/33 (45.4)	—^¶^/35 (—^¶^)	—^¶^/33 (—^¶^)	—^¶^/27 (—^¶^)
White	3,083/3,604 (85.5)	1,005/3,738 (26.9)	669/3,755 (17.8)	208/4,054 (5.1)	334/3,771 (8.9)	49/3,124 (1.6)
Hispanic or Latino	2,307/2,514 (91.8)	711/2,627 (27.1)	646/2,626 (24.6)	154/2,930 (5.3)	219/2,641 (8.3)	62/2,338 (2.7)
Multiracial and other	253/299 (84.6)	84/312 (26.9)	57/313 (18.2)	15/343 (4.4)	30/316 (9.5)	—^¶^/256 (—^¶^)
Missing	30/32 (93.8)	6/34 (17.7)	—^¶^/34 (—^¶^)	—^¶^/38 (—^¶^)	—^¶^/34 (—^¶^)	—^¶^/30 (—^¶^)
**Population density^††^**						
Large central metropolitan	2,892/3,278 (88.2)	866/3,422 (25.3)	750/3,418 (21.9)	168/3,738 (4.5)	301/3,442 (8.7)	72/2,927 (2.5)
Large fringe metropolitan	1,599/1,843 (86.8)	486/1,910 (25.5)	414/1,917 (21.6)	97/2,061 (4.7)	177/1,922 (9.2)	28/1,624 (1.7)
Medium metropolitan	1,603/1,801 (89.0)	517/1,858 (27.8)	377/1,861 (20.3)	108/2,053 (5.3)	149/1,869 (8.0)	26/1,619 (1.6)
Small metropolitan	580/671 (86.4)	208/709 (29.3)	136/711 (19.1)	50/782 (6.4)	74/717 (10.3)	9/592 (1.5)
Micropolitan	566/628 (90.1)	198/645 (30.7)	126/649 (19.4)	31/709 (4.4)	59/650 (9.1)	9/570 (1.6)
Noncore	310/339 (91.5)	117/355 (33.0)	57/356 (16.0)	15/384 (3.9)	22/356 (6.2)	7/317 (2.2)
Missing	243/268 (90.7)	98/279 (35.1)	58/282 (20.6)	10/321 (3.1)	15/283 (5.3)	0/244 (—)
**EHE jurisdiction^§§^**						
Yes	3,853/4,323 (89.1)	1,205/4,511 (26.7)	953/4,510 (21.1)	213/4,943 (4.3)	383/4,538 (8.4)	92/3,909 (2.4)
No	3,940/4,505 (87.5)	1,285/4,667 (27.5)	965/4,684 (20.6)	266/5,105 (5.2)	414/4,701 (8.8)	59/3,984 (1.5)
**Recruitment source^¶¶^**						
LGBTQ+ dating app	4,810/5,494 (87.6)	1,364/5,740 (23.8)	1,251/5,741 (21.8)	374/6,266 (6.0)	508/5,776 (8.8)	98/4,885 (2.0)
Other dating app or website	152/186 (81.7)	58/189 (30.7)	33/190 (17.3)	2/211 (1.0)	8/190 (4.2)	—^¶^/154 (—^¶^)
Social media	486/550 (88.4)	204/566 (36.0)	108/571 (18.9)	15/634 (2.4)	31/572 (5.4)	12/487 (2.5)
Other partner promotions	1,038/1,128 (92.0)	399/1,170 (34.1)	254/1,171 (21.7)	46/1,270 (3.6)	114/1,177 (9.7)	17/1,047 (1.6)
Search engine marketing or web search	630/674 (93.5)	199/688 (28.9)	139/693 (20.0)	15/771 (2.0)	68/693 (9.8)	13/635 (2.1)
Direct link	425/510 (83.3)	157/525 (29.9)	67/528 (12.7)	15/580 (2.6)	43/530 (8.1)	—^¶^/427 (—^¶^)
Other	27/30 (90.0)	12/31 (38.7)	8/31 (25.8)	—^¶^/36 (—^¶^)	—^¶^/31 (—^¶^)	—^¶^/27 (—^¶^)
Missing	225/256 (87.9)	97/269 (36.1)	58/269 (21.6)	12/280 (4.3)	24/270 (8.9)	—^¶^/231 (—^¶^)
Priority audience***						
No^†††^	1,701/1,987 (85.6)	598/2,051 (29.2)	352/2,062 (17.1)	68/2,285 (3.0)	173/2,072 (8.4)	26/1,719 (1.5)
Yes (all)	4,870/5,469 (89.1)	1,504/5,685 (26.5)	1,234/5,691 (21.7)	340/6,144 (5.5)	503/5,716 (8.8)	104/4,938 (2.1)
Yes, cisgender MSM	3,954/4,437 (89.1)	1,149/4,627 (24.8)	1,033/4,633 (22.3)	323/4,993 (6.5)	418/4,655 (9.0)	92/4,014 (2.3)
Yes, Hispanic cisgender MSM	1,276/1,383 (92.3)	370/1,446 (25.6)	363/1,444 (25.1)	100/1,578 (6.3)	113/1,451 (7.8)	38/1,293 (2.9)
Yes, Black or African American cisgender MSM	524/580 (90.3)	146/611 (23.9)	148/612 (24.2)	48/661 (7.3)	56/616 (9.1)	16/534 (3.0)
Yes, transgender women or transfeminine	163/178 (91.6)	56/186 (30.1)	51/184 (27.7)	11/206 (5.3)	21/186 (11.3)	6/165 (3.6)
Yes, Black or African American cisgender women	753/854 (88.2)	299/872 (34.3)	150/874 (17.2)	—^¶^/945 (—^¶^)	64/875 (7.3)	6/759 (0.8)
Unable to determine audience because of missing information	1,222/1,372 (89.1)	388/1,442 (26.9)	332/1,441 (23.0)	71/1,619 (4.4)	121/1,451 (8.3)	21/1,236 (1.7)
Missing information but recruited from LGBTQ+ dating app**^§§§^**	879/998 (88.1)	264/1,050 (25.1)	232/1,050 (22.1)	55/1,182 (4.7)	86/1,059 (8.1)	16/890 (1.8)

**Abbreviations:** AI/AN = American Indian or Alaska Native; EHE = Ending the HIV Epidemic in the US initiative; LGBTQ+ = lesbian, gay, bisexual, transgender, or queer-plus; MSM = men who have sex with men; NH/PI = Native Hawaiian or Pacific Islander; PrEP = HIV preexposure prophylaxis; STI = sexually transmitted infection.

* For each outcome, the number (no.) reporting a given outcome is divided by the total (No.) who completed a question about a given outcome on either a 10- or 60-day follow-up survey, stratified by characteristics in each row and reported as row percentages. When stratification by demographic or geographic groupings led to a numerator (no.) with at least one but fewer than five participants, numerator (no.) and percentages are suppressed.

^†^ One response missing for age; for this category total No. = 8,827.

^§^ Gender identity is a composite of reported sex at birth and current gender identity. Cisgender men and cisgender women reported a current gender identity that aligned with their reported sex at birth. Persons who stated their gender identity was transgender man or transmasculine or who reported female sex at birth and current gender of man were classified as transgender men. Persons who stated their gender identity was transgender woman or transfeminine or who reported male sex at birth and current gender of woman were classified as transgender women. Another gender identity includes persons who identified as nonbinary, gender nonconforming, genderqueer, gender fluid, or another gender.

^¶^ Dashes indicate numerator (no.) and percentage suppressed (at least one participant but fewer than five).

** Persons of Hispanic or Latino (Hispanic) origin might be of any race but are categorized as Hispanic; all racial groups are non-Hispanic. Other categories reported as a single race or as multiracial when more than one race was reported.

^††^ Zip codes associated with self-test shipping addresses were assigned to county of residence and then summarized using the National Center for Health Statistics population density categorizations. https://www.cdc.gov/nchs/data_access/urban_rural.htm

^§§^ Zip codes associated with self-test shipping addresses were assigned to county of residence and then categorized based on whether or not that county is included in one of the EHE jurisdictions. https://www.cdc.gov/ehe/php/jurisdictions-plans/

^¶¶^ The program ordering portal includes analytics that can categorize the source of recruitment to the website. These broad categories include LGBTQ+ dating app (dating apps for both gay and bisexual persons), other dating app or website (dating apps for other men and women), social media (e.g., Facebook and Instagram), other partner promotions (planned promotional campaigns by CDC partners in the Let’s Stop HIV together social media campaign [https://www.cdc.gov/stophivtogether/partnerships/toolkit/hiv-testing-my-way.html]), search engine marketing or web search, or direct link, when the link to the program was typed directly into an Internet browser.

*** For the first year, program marketing was developed for three priority audiences disproportionately affected by HIV: gay, bisexual, and other MSM; transgender women (of any race); and cisgender Black or African American (Black) women. For MSM, imagery was designed specifically to appeal to Black and Hispanic men, but messages were placed on social media and dating apps inclusive of all MSM.

^†††^ Transgender men and other gender-diverse persons, cisgender women who did not identify as Black, and men who only reported female or gender-diverse partners were not considered to have been among the priority audiences for marketing the program during the period covered by the report.

^§§§^ Persons who did not provide enough demographic or sexual behavior information but could be categorized as arriving at the ordering website from an LGBTQ+ dating app are also considered to have been reached by marketing efforts for priority audiences.

## Discussion

In its first year, TTMH distributed approximately 440,000 tests, which exceeded the program’s initial expectations of 200,000 for that year as well as the number reported for all other CDC-funded test sites considered nonclinical settings in 2021, combined (*8*). The percentage of persons reporting new positive HIVST results in TTMH was also approximately twice as high as that reported for all CDC-funded HIV testing in in-person settings (*8*). These data demonstrate that providing HIV self-tests was an effective option to reduce barriers for persons who are not otherwise receiving testing in clinic- or community-based settings. The program reached many persons who had not previously received testing for HIV as well as persons who had not received testing during the previous year, even among populations most affected by HIV. Nearly all persons who ordered from TTMH used an HIVST themselves, and approximately one quarter gave an HIVST to others, thereby extending the reach of the program. Within a relatively short time after ordering from TTMH, approximately 12% of participants sought out additional HIV and STI services, which demonstrates that mailed HIVSTs have not only the potential to increase HIV testing, but also might lead to additional important prevention and care-seeking behaviors.

The outcomes achieved by TTMH were the result of partnerships with LGBTQ+ dating apps (led by BHOC) and advertising implemented through CDC’s Let’s Stop HIV Together campaign.*** These efforts included marketing to populations disproportionately affected by HIV, including geographically based promotions within EHE jurisdictions. Tailored approaches helped the program address health equity by specifically attempting to reach populations disproportionately affected by HIV, including MSM (especially Black and Hispanic MSM), Black cisgender women, and transgender women. In addition, the program reached groups that included very high percentages of persons who reported never having received testing for HIV before they accessed the program, including young adults, transgender men, and other gender-diverse persons. Reaching these populations with HIV testing is critical to ensuring equitable access to care and prevention services. These populations might experience increased barriers to traditional in-person HIV testing methods, such as travel time and distance, cost, medical mistrust, and stigma. Mailed HIVSTs remove some of these barriers and thereby have the potential to expand equitable access to HIV testing.

### Limitations

The findings in this report are subject to at least two limitations. First, only 63.2% of all participants (138,698 of 219,360) provided sufficient demographic and sexual behavior information to permit ascertainment of whether they belonged to a priority audience. However, 73.0% of participants (22,572 of 30,925) who did not provide this information were recruited from LGBTQ+ dating apps, which suggests that those with missing demographic or sex behavior data are likely members of a priority audience. Combined, approximately two thirds (67.9%) of first-time orders were classified as being from priority audiences, but this might not directly correlate with their risk for acquiring HIV. Second, compared with in-person HIV testing programs, HIV self-testing programs face additional challenges of documenting traditional testing outcomes, including laboratory testing and subsequent HIV care or prevention services. TTMH, the Let’s Stop HIV Together campaign, and the materials in the HIVST packaging offer multiple resources to help users interpret their HIVST results and access services after testing; data from CDC-funded self-testing research has indicated that persons who use self-tests seek follow-up care at rates similar to those of persons using community-based testing (*1*). However, the low response rate for the 10- and 60-day follow-up surveys limits the data available to evaluate who is accessing these services and might have introduced bias in the evaluation of the program if, for example, persons who completed the follow-up survey were more likely to have had a positive self-test result or to have sought additional services. The low response to the follow-up surveys suggests a need to identify other supplemental evaluation approaches, such as cross-sectional surveys of randomly selected participants. In addition, health care providers can document patient-reported previous HIVST use when providing HIV testing to their patients; the HIV surveillance system case report form^†††^ has been updated to document use of HIV self-tests among persons with newly diagnosed HIV infections.

### Implications for Public Health Practice

HIV self-testing is, and will continue to be, an important means for increasing awareness of HIV status and facilitating access to HIV prevention and care. Clinicians, community organizations, and public health officials need to be aware of HIVST programs, initiate discussions about HIV testing conducted outside their clinics or offices, and initiate follow-up services for persons who report a positive or negative HIVST result.

## Footnotes

* https://www.cdc.gov/nchhstp/director-letters/launch-of-together-takemehome.html

†
https://www.cdc.gov/stophivtogether/index.html

§ In the first year, program marketing was developed for MSM; transgender women (of any race); and cisgender Black women. For MSM, imagery was designed specifically to appeal to Black and Hispanic or Latino (Hispanic) men, but messages were placed on social media and dating apps inclusive of all MSM. In this report, these population groups are referred to as priority audiences for program marketing efforts.

¶ https://together.takemehome.org** For persons who ordered from the program more than once, only demographic data reported when the first order was placed are considered.

†† Based on combined responses from one or both of the 10- and 60-day follow-up surveys. When stratification by demographic or geographic groupings led to numerators with at least one but fewer than five participants, numerator and percentages are suppressed. Strata with zero program users in the numerator are reported as zero percent.

§§ 45 C.F.R. part 46.102(l)(2), 21 C.F.R. part 56; 42 U.S.C. Sect. 241(d); 5 U.S.C. Sect. 552a; 44 U.S.C. Sect. 3501 et seq.

¶¶ Part of the marketing strategy included using ads that ran in counties and states that provide geographic focus for the EHE initiative. https://www.cdc.gov/ehe/php/jurisdictions-plans/

***
https://www.cdc.gov/stophivtogether/partnerships/toolkit/hiv-testing-my-way.html

†††
https://www.cdc.gov/hiv/pdf/guidelines/cdc-hiv-adult-case-report-form-2023.pdf
